# Seeing burnout coming: early signs and recognition strategies in health professionals

**DOI:** 10.3389/fpubh.2025.1721220

**Published:** 2025-11-26

**Authors:** Stefanos Karakolias

**Affiliations:** Department of Nursing, Democritus University of Thrace, Alexandroupolis, Greece

**Keywords:** burnout syndrome, early recognition, warning signs, health professionals, occupational stress, emotional exhaustion, preventive strategies

## Abstract

Burnout syndrome is a pervasive psychological condition arising from prolonged occupational stress. Health professionals, particularly those in high-stakes environments, are especially vulnerable due to chronic workplace pressures, high job demands, and limited resources. While the prevalence and risk factors of burnout are well-documented, early warning signs often go unrecognized until the condition becomes chronic. This review synthesizes current evidence from 45 studies published between 2010 and 2025 on the early indicators and recognition strategies of burnout, introducing a three-domain framework for proactive detection. The first domain involves intrapersonal indicators, including emotional, cognitive, and physical symptoms such as persistent fatigue, impaired concentration, poor sleep quality, and physical complaints. The second domain focuses on interpersonal indicators, reflected in depersonalization, irritability, reduced empathy, and expressions of dissatisfaction. The third domain comprises occupational manifestations, ranged from absenteeism, tardiness, and declining job performance to unhealthy overcommitment despite apparent productivity. Effective strategies involve systematic monitoring of behavioral changes, validated assessment tools, predictive analytics, workplace surveys, and tracking of physiological stress markers. Organizational interventions, including supportive leadership, resilience and emotional intelligence training, workload management, and fostering a positive work culture, are crucial. This review emphasizes early recognition as a cornerstone for preventive strategies and underscores the need for evidence-based, proactive policies to safeguard healthcare professionals’ wellbeing and optimize occupational health outcomes.

## 1 Introduction

Burnout syndrome, commonly referred to as burnout, is a psychological condition resulting from prolonged exposure to occupational stressors and is characterized by three core dimensions: emotional exhaustion, depersonalization, and a lack of personal accomplishment. Emotional exhaustion refers to the feeling of being emotionally overextended and depleted of one’s emotional resources. Depersonalization involves developing a cynical attitude and feelings of detachment from one’s job and the people involved. Finally, a reduced sense of personal accomplishment entails feelings of incompetence and lack of success in one’s work (1, 2).

This condition frequently affects individuals engaged in professions that demand significant personal involvement, such as educators, military personnel, and health professionals. Such individuals face chronic workplace stress, where they encounter high demands paired with low resources, leading to burnout syndrome (2, 3). While burnout is predominantly linked to job-related stress, some argue for a broader understanding that considers it within a multi-domain frame, recognizing it as a stress-related condition not entirely confined to the workplace (4).

Burnout is a critical concern among health professionals, with prevalence rates varying across settings and regions. A meta-analysis conducted during the COVID-19 pandemic reported a pooled burnout prevalence of 43.6% among medical staff across 46 countries (5). In the United States, a study of the Veterans Health Administration found annual burnout rates among healthcare workers of 30.4% in 2018, 31.3% in 2019, 30.9% in 2020, 35.4% in 2021, 39.8% in 2022, and 35.4% in 2023, with primary care physicians experiencing even higher rates, ranging from 46.2% in 2018 to 57.6% in 2022 (6). Similarly, in Canada, 78.7% of public health workers were found to experience burnout (7). Burnout rates are particularly pronounced in high-stress environments such as intensive care units (ICUs), where prevalence can reach up to 80%, driven by factors such as prolonged work hours, organizational pressures, and the emotional demands of end-of-life care (8).

Similar trends have been observed among early career mental health professionals, who often experience high levels of emotional exhaustion and depersonalization (3). Stressors such as workplace bullying, lack of support, high job stress, and inadequate staffing are significant predictors of burnout in various healthcare settings (9). Furthermore, significant geographical variations exist, with burnout prevalence in Middle Eastern countries being exacerbated by unique regional challenges, such as exposure to violence and terrorism (10). The COVID-19 pandemic further intensified these challenges, increasing emotional exhaustion among healthcare workers due to factors such as prolonged personal protective equipment use and increased work pressure (11).

In essence, burnout arises from a mismatch between individual traits and workplace or organizational factors. The ramifications of this pervasive issue are substantial, manifesting as elevated staff turnover, decreased job satisfaction, and significant impairments in the quality and safety of patient care, which are especially consequential in the context of a global shortage of healthcare professionals (6, 12). Recognizing this, there is a call for comprehensive interventions targeting organizational reforms, improved support systems, and resilience-building strategies to address this issue. Important protective factors include supportive work environments, sufficient staffing, and resilience training, which can potentially mitigate burnout incidence (9, 11).

## 2 Determinants of burnout and the current research gap

Burnout among healthcare professionals is influenced by a range of determinants, often referred to as risk factors, encompassing psychological, organizational, and demographic domains. Emotional exhaustion, the central element of burnout, is closely linked to perceived stress and fear of contagion (13). During the COVID-19 pandemic, healthcare workers experienced heightened psychological stressors due to the high-stakes nature of their work environments (11). Workplace dynamics are also significant, as high levels of job stress, low job satisfaction, and inadequate communication are primary occupational risk factors contributing to burnout (9). Another risk factor is the work environment. Long working hours, shift work, and high patient acuity are significant organizational stressors contributing to burnout. These factors are particularly prevalent in intensive care units and emergency rooms, where the intensity and unpredictability of work are heightened (14, 15).

Existing research has underscored the influence of demographic factors on the risk of burnout, including sex, age, and occupational role. Female healthcare professionals, younger individuals, and frontline workers frequently report elevated burnout levels. Similarly, individuals with college degrees and those holding higher-ranking positions are at an increased risk because of augmented responsibilities and expectations (11, 16). Moreover, a positive organizational culture can act as a safeguard against burnout, whereas workplace bullying and inadequate organizational support tend to intensify it (9). Finally, external stressors, such as exposure to disasters or pandemics, which increase physical and mental workloads, contribute significantly to burnout. For instance, healthcare workers in settings affected by natural disasters or global health emergencies, such as COVID-19, experience heightened burnout levels (15, 17).

As previously indicated, extensive research has quantified burnout and examined its determinants; however, understanding the initial symptoms or warning signs that precede full-blown burnout remains limited. Notably, burnout was first conceptualized in the 1970s by psychoanalyst Herbert Freudenberger, who described it as a state of mental and physical exhaustion experienced by “helping” professionals. Freudenberger’s (18) model outlined a progression from excessive drive and ambition, through chronic stress, to full burnout. Focusing on early warning signs aligns with identifying symptoms in the intermediate stages of such models, before the condition becomes chronic, as these indicators often remain undetected until burnout is well established (19). Highlighting these preliminary signs enables a proactive approach to prevention, emphasizing the need to examine emerging stressors and their potential to escalate into severe burnout. Strategies for timely recognition of early symptoms are therefore essential. This perspective is reinforced by major reviews, including the 2019 U.S. National Academy of Medicine report, which underscored the importance of organizational interventions in addressing what it described as a “public health crisis” among clinicians (20). In this context, the present review aims to synthesize existing evidence on the early manifestations of burnout and strategies for its early identification.

## 3 Methodology

This short review employed a narrative approach to synthesize current evidence on early signs and recognition strategies for burnout among health professionals. The literature search encompassed two major databases: PubMed and Scopus. The search strategy utilized combinations of key terms including “burnout,” “healthcare professionals,” “early signs,” “predictors,” “recognition,” and “prevention.” To ensure relevance and currency, the review focused on articles published in English between 2010 and 2025. The initial search yielded a substantial pool of 487 articles, which underwent a preliminary screening process based on titles and abstracts. This screening, along with the removal of duplicates and irrelevant articles, resulted in 68 full-text articles being assessed for eligibility. Following a thorough evaluation, 45 articles were ultimately selected for inclusion in the review based on their direct relevance to early burnout indicators and recognition strategies specific to healthcare settings. The final selection of articles encompassed a diverse range of scholarly works, including original research studies, systematic reviews, and expert commentaries. This varied selection was intentional, aiming to provide a comprehensive and multifaceted overview of the topic. By incorporating different types of academic literature, the review sought to capture both empirical findings and expert insights on early burnout recognition and prevention strategies in the healthcare sector.

## 4 Early signs of burnout

Early signs of burnout can be characterized as the initial symptoms or warning indicators that suggest an individual is beginning to experience burnout prior to its progression into a severe, chronic condition. Among healthcare professionals, these early behavioral indicators often present in subtle yet significant ways, signaling the potential onset of more serious issues if not addressed in a timely manner.

Maslach’s (21) multidimensional model conceptualizes job burnout as encompassing three dimensions: overwhelming exhaustion, feelings of cynicism and detachment from one’s work, and a perceived sense of ineffectiveness and failure. Similarly, the Job Demands-Resources (JD-R) model posits that a prolonged imbalance between job demands and available resources leads to strain reactions that affect both personal wellbeing and work performance (22). Furthermore, the Conservation of Resources (COR) theory suggests that continuous resource depletion triggers protective behaviors, such as withdrawal or absenteeism (23). Collectively, these frameworks support a multidimensional understanding of early burnout signs. In this context, this review identified and presents three types of signs: (1) intrapersonal signs, which reflect emotional, cognitive, and physical changes within the individual; (2) interpersonal signs, which are manifestations in the person’s relationships or interactions with others; and (3) occupational signs, which represent observable work-related behaviors indicating reduced engagement or functioning.

### 4.1 Intrapersonal signs

Emotional exhaustion is a primary indicator of burnout, characterized by individuals experiencing feelings of being overwhelmed and emotionally depleted by their work, often resulting in persistent fatigue that does not subside with rest (24–26). Individuals experiencing fatigue frequently demonstrate reduced cognitive function, including impaired concentration and attention (27). Concurrently, emotional exhaustion contributes to poor sleep quality. Unlike the transient tiredness that may result from a long shift, this burnout-related fatigue is often characterized by its persistence, where sleep and rest no longer feel restorative, further exacerbating the cycle of exhaustion (28, 29).

Physical fatigue is another manifestation of burnout, as emotional exhaustion often results in significant physical strain. This condition adversely affects physical mobility by reducing energy levels and motivation to engage in physical activities, thereby impacting overall health and functional capability (30, 31). Early stages of burnout can also coincide with heightened anxiety and depressive symptoms, as observed during high-stress periods, such as the COVID-19 pandemic (32). Generally, the onset of burnout may be marked by physical signs of stress, such as changes in appetite or sleep patterns, frequent headaches, and gastrointestinal issues (33).

### 4.2 Interpersonal signs

Burnout among healthcare professionals frequently manifests as depersonalization, characterized by a sense of detachment and a negative, cynical attitude toward patients or colleagues, which may initially manifest as withdrawal from work-related interactions (34–36) or a more generalized, intentional isolation from peers (37). A noticeable shift in interpersonal behavior often signals early burnout. This can manifest as uncharacteristic irritability and frustration, where an individual’s tolerance for social situations diminishes, complicating interactions with colleagues and patients in a way that deviates from their typical demeanor (38, 39). Elevated levels of emotional exhaustion similarly reduce empathy, leading to mechanical and detached interactions, particularly in caregiving professions such as healthcare (38, 40).

From another perspective, burnout can significantly impact an individual’s perceived competence and the value they place on their work, often resulting in expressions of dissatisfaction or “grumbling” (3, 41). This self-doubt is exacerbated by the emotional exhaustion and cynicism that characterize burnout, undermining self-confidence, and reducing perceived personal and collective efficacy (42, 43). Conversely, grumbling can be attributed to a sense of frustration over a perceived lack of support, overwhelming job demands, and insufficient resources, all of which contribute to the exhaustion and depersonalization experienced by those affected (44, 45).

### 4.3 Occupational signs

A decline in job performance is arguably the most prominent indicator of burnout, manifesting not only in attendance issues but also directly in the quality of care delivered to patients. This deterioration initially manifests as a combination of increased sick leave and absenteeism, as employees experiencing burnout contend with both physical and mental exhaustion (46). Additionally, there is a noticeable increase in tardiness and a decline in punctuality, as affected individuals struggle to arrive at work on time and maintain their usual productivity levels (39). This is further associated with the occurrence of uncharacteristic errors, forgetfulness regarding tasks, and difficulty concentrating (47). This decline may also be evident in patient-related metrics. Clinicians who previously demonstrated strong patient relationships may begin to receive atypical complaints or reduced satisfaction scores. Such deviations from prior performance levels may represent early indicators of burnout, reflecting intrapersonal changes such as irritability, frustration, and diminished empathy mentioned above.

While declining job performance is a common indicator, burnout can paradoxically occur among high-performing employees, a phenomenon observed even outside the health sector. In these cases, the occupational sign is not a drop in productivity but rather an unhealthy overcommitment to work at the expense of personal wellbeing. This can be distinguished from healthy dedication through its underlying drivers and consequences. Healthy dedication is typically balanced and sustainable, whereas unhealthy overcommitment is often characterized by an inability to disengage from work, such as staying late unnecessarily. This behavior, while appearing productive, can signify a problematic attachment to work tasks that hints at underlying emotional exhaustion and may represent an early stage of burnout before a noticeable decline in performance occurs (48, 49).

Furthermore, burnout exerts a substantial influence on teamwork and collaboration within organizations, either directly or by intensifying pre-existing challenges, such as workplace incivility and dissatisfaction (50). The extant literature underscores the deterioration of teamwork dynamics, as individuals experiencing emotional exhaustion are less inclined to engage effectively and collaborate with their colleagues (51). This deficiency may prompt employees to consider resigning or actually resign from their positions, thereby further undermining team cohesion and stability (52, 53).

These multifaceted early warning indicators, categorized within the intrapersonal, interpersonal, and occupational domains, are synthesized and presented in Table 1 for clear reference.

**Table 1:** Summary of early signs of burnout by domain.

Domain	Early signs
Intrapersonal	Persistent fatigue not relieved by rest Impaired concentration and attention Poor sleep quality Physical complaints (e.g., headaches, gastrointestinal issues) Heightened anxiety and depressive symptoms
Interpersonal	Detachment and cynical attitude (depersonalization) Irritability and frustration Reduced empathy Expressions of dissatisfaction or “grumbling”
Occupational	Decline in job performance (increased sick leave, absenteeism, tardiness) Uncharacteristic errors and forgetfulness Unhealthy overcommitment to work Deterioration of teamwork and collaboration Increased intention to resign or actual resignation

## 5 Strategies for early recognition of burnout

A comprehensive approach is essential to effectively identify early indicators of burnout. The strategies outlined herein are supported by the existing literature.

Healthcare organizations should systematically monitor behavioral changes among employees. Indicators such as absenteeism, tardiness, and a decline in punctuality may serve as early signs of burnout, suggesting that employees are either physically unwell or mentally disengaged from their work (54). In recent years, predictive analytics, including Neural Networks and Deep Learning algorithms, have been employed to forecast patterns in employee attendance behaviors (55).

Subsequently, healthcare organizations should conduct workplace surveys and develop additional feedback mechanisms. These surveys should focus on job satisfaction, stress levels, and workplace engagement. Surveys can reveal underlying issues related to burnout symptoms, such as cognitive weariness, isolation, and reduced performance (56). Additional feedback mechanisms include the use of technological tools to track physiological indicators, such as heart rate and sleep patterns, to monitor signs of stress and burnout (57). A best practice observed in hospitals across the United States, particularly in various orthopedic departments, is the frequent use of the Maslach Burnout Inventory (MBI) to objectively assess burnout levels among the medical staff. This tool aids in the routine identification of symptoms, such as emotional exhaustion and depersonalization (35).

Implementing organizational interventions is vital. First, it is crucial to educate managers and leaders on identifying the early signs of burnout and adopting supportive management practices. This training should include workshops on stress management and fostering supportive work culture (58). Additionally, creating an environment that encourages open discussion of stress and workload issues is essential. Establishing strong support systems and conducting regular check-ins can help identify and address burnout before it worsens (59). This also involves nurturing a workplace culture that prioritizes the wellbeing of employees. Promoting a culture in which staff feel valued and recognized for their contributions can reduce workplace stress and enhance job satisfaction (60). Encouraging skill development is also advantageous because it can boost job engagement and reduce burnout (61).

Building on leadership and cultural initiatives, it is equally important to implement programs for employees that focus on cultivating emotional intelligence and resilience in the workplace. These initiatives support employees in effectively managing stress, enhancing interpersonal relationships, and maintaining job satisfaction, thereby preventing the progression of burnout (62, 63). A notable example can be observed in China, where clinical therapists working in tertiary psychiatric hospitals have engaged in resilience training programs aimed at alleviating stress and preventing burnout in the workplace. These programs have proven effective in enhancing emotional intelligence and stress management in high-pressure environments (64). Another exemplary practice is found in mental health settings across the United States, where there is advocacy for integrating burnout recognition and management strategies into medical curricula (3).

Complementing these psychological and educational strategies, it is imperative to adjust workload distribution and ensure that job roles and expectations are realistic and clearly articulated. Providing flexibility and resources to support a healthy work-life balance can substantially mitigate the risk of burnout (65, 66). Likewise, addressing staffing shortages and managing workloads to prevent excessive stress and burnout are essential. Maintaining adequate staffing levels and balanced workloads can enhance job satisfaction and alleviate stress (60, 67).

Finally, it is essential to establish continuous monitoring and evaluation mechanisms to assess the effectiveness of the aforementioned interventions and to refine strategies in response to staff feedback and evolving organizational demands. Such an adaptive approach ensures that implemented measures remain contextually relevant, evidence-based, and effective in mitigating burnout (68). Through the integration of these strategies, healthcare organizations can develop a comprehensive and sustainable framework that addresses the underlying determinants of burnout while fostering a supportive and health-promoting work environment.

To provide a clear, actionable framework, these multifaceted strategies are synthesized and tiered by stakeholder level in Table 2, outlining responsibilities from the individual to the organizational level.

**Table 2:** Tiered strategies for early burnout recognition.

Level	Strategy	Description and rationale
Individual	Self-monitoring & skill development	Employees engage in self-reflection on wellbeing and participate in programs to build emotional intelligence and resilience to manage stress effectively.
Peer	Supportive culture & open dialogue	Fostering an environment where colleagues feel safe to discuss stress and workload issues, providing mutual support and encouraging open communication.
First-line supervisor	Supportive leadership & early identification	Managers are trained to recognize early behavioral signs of burnout (e.g., absenteeism, tardiness) and conduct regular check-ins to address issues proactively.
Organizational	Systematic monitoring	Implement workplace surveys, predictive analytics, and feedback mechanisms to track stress levels, job satisfaction, and physiological stress markers.
	Workload management	Ensure realistic job expectations, adequate staffing, and balanced workloads to prevent excessive stress and support a healthy work-life balance.
	Cultural & educational initiatives	Promote a positive work culture, integrate burnout management into training, and provide resources for skill development and resilience.
	Continuous evaluation	Establish mechanisms to continuously monitor and refine the effectiveness of anti-burnout interventions based on staff feedback and evolving needs.

## 6 Discussion

### 6.1 Cultural and contextual considerations

Cultural norms and values can significantly impact how burnout is expressed and perceived. In collectivist cultures, healthcare professionals may be less likely to report personal distress due to concerns about group harmony. Conversely, in individualistic cultures, personal wellbeing might be more openly discussed (69). These cultural differences necessitate tailored approaches to burnout early recognition and intervention.

While this review presents a general framework for recognizing early signs of burnout, it is crucial to acknowledge that its application may require adaptation to suit diverse healthcare contexts. Factors such as organizational structure, professional hierarchies, and local health policies can influence how burnout manifests and is addressed. Specifically, applying burnout recognition strategies in low-resource settings presents unique challenges. Healthcare professionals in these contexts often face additional stressors such as resource scarcity, higher patient loads, and limited support systems (70). Strategies for burnout recognition in such environments may require a more nuanced approach, prioritizing cost-effective and easily implementable methods.

### 6.2 Limitations

While this review synthesizes the early manifestations of burnout, it is important to recognize the existing limitations in the literature regarding their predictive validity. A significant research gap remains in understanding the precise timing and progression of these early symptoms. Specifically, current evidence has yet to determine how far in advance these signs emerge before burnout becomes a chronic condition, nor has it established their sensitivity and specificity as diagnostic markers. Although certain early predictors have been proposed, further research is required to validate a comprehensive early warning system. Developing and empirically validating such a system would represent a critical advancement, enabling interventions that are both timely and targeted, thereby improving outcomes for healthcare professionals.

The proposed three-domain framework for the proactive detection of burnout has an inherent limitation. In particular, the intrapersonal signs domain, which includes emotional exhaustion, anxiety, depressive symptoms, fatigue, and physical complaints, overlaps substantially with other clinical conditions such as major depressive disorder, anxiety disorders, and chronic fatigue syndrome. A key distinguishing factor often lies in context: burnout symptoms are typically work-related and may improve with time away from work, whereas clinical depression tends to persist across life domains (71). Nevertheless, given the considerable symptomatic overlap and potential for comorbidity, early warning signs should not be dismissed as mere “work stress.” When such symptoms are persistent, severe, or significantly impair overall functioning, referral for a comprehensive clinical evaluation is warranted to ensure accurate diagnosis and appropriate management of any underlying or co-existing conditions.

### 6.3 Directions for future research

Given the importance of recognizing early warning signs prior to examining burnout in its fully developed state, the focus of this review is to highlight the existing gap in predictive understanding rather than to draw upon an established body of longitudinal evidence. The recommendations to monitor behavioral indicators such as absenteeism and to employ standardized instruments like the MBI are grounded in well-documented correlational research. In contrast, approaches involving predictive analytics constitute an emerging area of inquiry whose effectiveness remains to be empirically validated. Accordingly, future research should prioritize longitudinal designs to address these evidence gaps, verify the predictive validity of early indicators, and advance the field from correlational to causal understanding. Such efforts will enable the development of interventions supported by robust, high-quality evidence.

## 7 Conclusion

This short review highlights the critical importance of early detection and intervention strategies for burnout among health professionals, carrying significant implications for Occupational Health and Safety (OHS) policy. It emphasizes the multifaceted nature of burnout, spanning intrapersonal, interpersonal, and occupational domains, and calls for integrated, proactive approaches. From an OHS perspective, organizations should implement systematic monitoring of behavioral indicators such as absenteeism, incorporate predictive analytics and regular job satisfaction surveys, and ensure that managers are trained to recognize early warning signs. Policies should also foster supportive work cultures through mandatory stress management training, open dialogue about workload issues, and the establishment of robust support systems. Additionally, promoting skill development, emotional intelligence, and resilience training should be integral to workforce wellbeing initiatives. Ensuring balanced workloads, clear job expectations, and regulated rest periods is essential for sustainable occupational health. Finally, anti-burnout measures must be dynamic, regularly reviewed and refined based on staff feedback and organizational change, to safeguard both the wellbeing and productivity of healthcare professionals.
